# Mapping the global landscape of chikungunya rapid diagnostic tests: A scoping review

**DOI:** 10.1371/journal.pntd.0010067

**Published:** 2022-07-25

**Authors:** José Moreira, Patrícia Brasil, Sabine Dittrich, André M. Siqueira

**Affiliations:** 1 Laboratório de Pesquisa Clínica em Doenças Febris Agudas, Instituto Nacional de Infectologia Evandro Chagas, Fundação Oswaldo Cruz, Rio de Janeiro, Brazil; 2 Departamento de Ensino & Pesquisa, Instituto Nacional de Cardiologia, Rio de Janeiro, Brazil; 3 Malaria & Fever Department, Foundation for Innovative New Diagnostics, Geneva, Switzerland; 4 Nuffield Department of Medicine, University of Oxford, Oxford, United Kingdom; Center for Disease Control and Prevention, UNITED STATES

## Abstract

**Background:**

Chikungunya (CHIKV) is a reemerging arboviral disease and represents a global health threat because of the unprecedented magnitude of its spread. Diagnostics strategies rely heavily on reverse transcriptase-polymerase chain reaction (RT-PCR) and antibody detection by enzyme-linked Immunosorbent assay (ELISA). Rapid diagnostic tests (RDTs) are available and promise to decentralize testing and increase availability at lower healthcare system levels.

**Objectives:**

We aim to identify the extent of research on CHIKV RDTs, map the global availability of CHIKV RDTs, and evaluate the accuracy of CHIKV RDTs for the diagnosis of CHIKV.

**Eligibility criteria:**

We included studies reporting symptomatic individuals suspected of CHIKV, tested with CHIKV RDTs, against the comparator being a validated laboratory-based RT-PCR or ELISA assay. The primary outcome was the accuracy of the CHIKV RDT when compared with reference assays.

**Sources of evidence:**

Medline, EMBASE, and Scopus were searched from inception to 13 October 2021. National regulatory agencies (European Medicines Agency, US Food and Drug Administration, and the Brazilian National Health Surveillance Agency) were also searched for registered CHIKV RDTs.

**Results:**

Seventeen studies were included and corresponded to 3,222 samples tested with RDTs between 2005 and 2018. The most development stage of CHIKV RDTs studies was Phase I (7/17 studies) and II (7/17 studies). No studies were in Phase IV. The countries that manufacturer the most CHIKV RDTs were Brazil (*n* = 17), followed by the United States of America (*n* = 7), and India (*n* = 6). Neither at EMA nor FDA-registered products were found. Conversely, the ANVISA has approved 23 CHIKV RDTs. Antibody RDTs (*n* = 43) predominated and demonstrated sensitivity between 20% and 100%. The sensitivity of the antigen RDTs ranged from 33.3% to 100%.

**Conclusions:**

The landscape of CHIKV RDTs is fragmented and needs coordinated efforts to ensure that patients in CHIKV-endemic areas have access to appropriate RDTs. Further research is crucial to determine the impact of such tests on integrated fever case management and prescription practices for acute febrile patients.

## Introduction

Chikungunya—a reemerging arboviral disease caused by Chikungunya virus (CHIKV)—is transmitted by mosquitoes of the *Aedes* species, specifically *Aedes aegypti*, *Aedes albopictus*, and *Aedes polynesiensis* [1]. The disease is characterized by the classic triad of debilitating polyarthralgia, high-grade fever, and myalgia [1]. During the past years, we have seen an unprecedented magnitude of the disease spreading across the globe (i.e., 106 countries/territories reported autochthonous or travel-related transmission), affecting millions of people in the Americas, Asia, the Indian subcontinent, Europe, and in the Pacific islands [2].

One of the challenges imposed by CHIKV has been the correct identification of suspected individuals in the context of co-circulation of other arboviruses that present similarly in tropical regions [3]. Laboratory diagnosis has been mainly focused on either RNA or virus-specific antibody detection through reverse transcriptase-polymerase chain reaction (RT-PCR) and enzyme-linked Immunosorbent assay (ELISA) technique, respectively. However, such diagnostic technologies require complex instrumentation and are not easy to perform outside sophisticated laboratories in urban settings where trained personnel are available. Therefore, these tests are not accessible or affordable to patients at the lower healthcare system levels, where most CHIKV outbreaks occur. In contrast, rapid diagnostic tests (RDTs) promise to overcome some of these challenges by bridging many gaps along the diagnostic test pathway in CHIKV-endemic areas.

RDTs have become available for detecting CHIKV and are reported to have variable performance and operational characteristics [4–6]. Much remains unknown regarding how these tests increase the efficiency of the health systems if introduced appropriately, how acceptable they are for patients and health care providers, and how cost-effective they are, given the poor state of many countries’ economies primarily impacted by CHIKV. Thus, we aim to (i) identify the extent of research on CHIKV RDTs; (ii) provide a comprehensive landscape of CHIKV RDTs available globally; (iii) evaluate the performance of CHIKV RDTs for the diagnosis of CHIKV in symptomatic individuals when compared with a reference standard; and (iv) identify knowledge gaps and further research related to CHIKV RDTs.

## Methods

We followed the PRISMA Extension for Scoping Reviews (Prisma-ScR) guidance from the EQUATOR (Enhancing the QUAlity and Transparency Of health Research) Network [7]. The Prisma-ScR checklist is available in S1 PRISMA Checklist.

### Eligibility criteria

Search terms were based on a PICO (population, intervention, comparator, and outcome) framework. The population encompassed symptomatic febrile individuals suspected of CHIKV infection. The intervention used CHIKV RDTs, either in developmental or commercially available, to diagnose CHIKV infection, with the comparator being a validated laboratory-based RT-PCR or ELISA assay. The primary outcome was the accuracy of the CHIKV RDT when compared with reference assays.

Articles were excluded if (i) the studies were reviews, case reports, or opinion articles; (ii) the studies evaluated the performance of reverse transcription loop-mediated isothermal amplification (RT-LAMP) assays; (iii) the studies were related to an outbreak investigation without the evaluation of the accuracy of CHIKV RDTs; (iv) the studies used an inappropriate study population (asymptomatic individuals); (v) the studies described inappropriate reference assays to assign true positive/true negative status to study samples; and (vi) studies that were related to other arboviruses.

### Operational definitions

- CHIKV RDT was defined as a rapid (≤60 min) point-of-care (POC) assay that requires minimal instrumentation to provide actionable results.
- We classified the stage of CHIKV RDT assay development in 4 phases: Phase I, which consist of the prototype evaluation process; Phase II evaluation under ideal conditions using convenience or archived samples; Phase III evaluations under ideal conditions assessing the performance and operation characteristics of the index test in a target population; and Phase IV, which are assessments of the impact of diagnostics on the prevalence of infection, the incidence of infection, or incidence of complications.

### Information sources

Medline, EMBASE, and Scopus electronic databases were searched from inception to 13 October 2021 to identify relevant publications in peer-reviewed journals as original scientific research. Additional studies were identified through manual searches of the reference lists of identified papers. The electronic database search was supplemented by searching at major tropical medicine conference abstracts repositories and the manufacturer’s official website to seek relevant published reports. The final search results were exported into Mendeley to manage citations identified.

In order to provide a comprehensive assessment of diagnostic products that are in the developmental phase and commercialization, we conducted searches in national regulatory agencies (i.e., European Medicines Agency, US Food and Drug Administration, and the Brazilian National Health Surveillance Agency) websites looking for registered CHIKV RDTs and a free search through the Google search engine.

### Search

The search in Medline was performed using the following terms: chikungunya or “chikungunya virus” or “chikungunya fever” and “rapid diagnostic test” or “rapid test”. There was no language or time restriction. After deleting duplicates, the literature review group systematically screened the title, abstract, and full text of each study’s inclusion and exclusion criteria.

### Data charting process

Data were extracted independently from the selected studies by 2 authors and recorded into a standard form designed for this study. Discrepancies were resolved by mediation and discussion with other reviewers if needed. The standardized data abstraction tool captured the relevant information on key study characteristics and detailed information on all metrics used to estimate the accuracy of the CHIKV RDTs. Key variables that were systematically extracted include the year of investigation, geographical location, study design, type of RDT assay, time of illness onset to testing, reference assay, sample size, and diagnostic accuracy parameters (if available). If a study evaluated more than 1 RDT assay, we extracted the data related to each assay type. When articles did not provide sufficient information on relevant data, we contacted the authors via email for additional information.

### Critical appraisal of individual sources of evidence

The quality of each diagnostic accuracy study was assessed following QUADAS-2 guidelines [8].

### Synthesis of results

Data from all studies were aggregated, and frequency statistics were run to describe the population across all studies. Tableau Desktop Professional Edition (Tableau software, LLC, version 2021.1.0, Seattle, Washington, United States) and GraphPad Prism (GraphPad Software, version 8.0, San Diego, California, US) were used to represent the evidence visually.

## Results

### Search results

The initial search identified 271 potential studies for evaluation (S1 PRISMA Flowchart). After duplicates were removed, a total of 185 citations were identified from searches of electronic databases. Based on the tile and the abstract, 96 were excluded, with 89 full-text articles retrieved and assessed for eligibility. The remaining 17 studies were considered eligible for this review (all apart from 1 reported diagnostic accuracy metric).

### Description of studies

A summary of the included studies is shown in Table 1. The main countries where the CHIKV patients were sourced were India (3/17 studies, 17.6%), Thailand (3/17 studies, 17.6%), Indonesia (2/17 studies, 11.7%), and Aruba (2/17 studies, 11.7%) (S1 Fig). CHIKV RDTs studies were Phases I (7/17 studies, 41.1%) and II (7/17 studies, 41.1%) in most included studies. Two studies were Phase III [4,9]. No study was Phase IV. Sample recruitment used case-control methodologies (13/17 studies, 76.4%), a prospective cohort design (3/17 studies, 17.6%), or described the development of a pilot RDT assay (1/17 studies, 5.8%) [10]. Description of the tested population and the setting where they were applied was almost absent in the studies.

**Table 1:** Characteristics of included studies evaluating Chikungunya antibody or antigen-based rapid diagnostic tests, 2005–2018.

First author, year [Reference]	Location	Study design	Assay	Assay’s phase of diagnostic development	Setting	Age (years)	Severity
**Reddy A and colleagues 2020 [22]**	Honduras and Colombia	Case-control	E1/E2-Antigen test	Phase I	ND	ND	ND
**Suzuki and colleagues 2020 [5]**	Aruba and Bangladesh	Case-control	E1-Antigen test	Phase I	ND	ND	ND
**Lee H and colleagues 2020 [11]**	ND	Case-control	ichroma Chikungunya virus (IgG/IgM)	Phase II	ND	ND	ND
**Kim WS and colleagues 2019 [12]**	ND	Case-control	Chikungunya IgM/IgG (GenBody)	Phase II	ND	ND	ND
**Wang R and colleagues 2019 [19]**	Colombia	Case-control	DENV IgG/IgM CHIKV IgG/IgM	Phase I	ND	18–74	ND
**Huits R and colleagues 2018 [6]**	Mauritius, Réunion, India, Thailand, French Polynesia, Aruba	Case-control	E1-Ag test	Phase I	ND	ND	ND
**Jain J and colleagues 2018 [21]**	India	Case-control	E1-Ag test	Phase I	ND	ND	ND
**Lee S and colleagues 2016 [10]**	ND	Development study	DENV IgG/IgM CHIKV IgG/IgM	Phase I	ND	ND	ND
**Burdino E and colleagues 2016 [13]**	Caribbean and Latin America	Prospective recruitment	OnSite Chikungunya IgM Combo Rapid test	-	ND	ND	ND
**Johnson BW and colleagues 2016 [18]**	ND	Case-control	OnSite CHIKV IgM Combo Rapid test SD BIOLINE Chikungunya IgM	Phase II	ND	ND	ND
**Okabayashi T and colleagues 2015 [20]**	Thailand, Laos, Indonesia, and Senegal	Case-control	E1-Ag test	Phase I	ND	ND	ND
**Prat CM and colleagues 2014 [14]**	ND	Case-control	SD BIOLINE Chikungunya IgM OnSite Chikungunya IgM Combo Rapid test	Phase II	ND	ND	ND
**Kosasih H and colleagues 2012 [15]**	Indonesia	Case-control	OnSite Chikungunya IgM Rapid test SD BIOLINE Chikungunya IgM test	Phase II	ND	ND	ND
**Arya SC and colleagues 2011 [16]**	India	Case-control	OnSite Chikungunya IgM Rapid test	Phase II	ND	ND	ND
**Yap G and colleagues 2010 [17]**	Singapore	Case-control	OnSite Chikungunya IgM Combo Rapid test	Phase II	ND	ND	Severe
**Rianthavorn P and colleagues 2010 [4]**	Thailand	Prospective recruitment	OnSite Chikungunya IgM Combo Rapid test	Phase III	ND	ND	ND
**Mistretta M and colleagues 2009 [9]**	Italy	Prospective recruitment	OnSite Chikungunya IgM Combo Rapid test	Phase III	ND	ND	ND

Phases of diagnostics developments are classified in 4 phases: Phase I, which consist of prototype evaluation process; Phase II evaluation under ideal conditions using convenience or archived samples; Phase III evaluations under ideal conditions assessing the performance and operation characteristics of product in target populations; and Phase IV, which are assessments of impact of diagnostics on prevalence of infection, incidence of infection, or incidence of complications.

ICT, immunochromatographic assay; IQR, interquartile range; ND, not described.

### Global availability of Chikungunya RDTs

**Fig 1:**
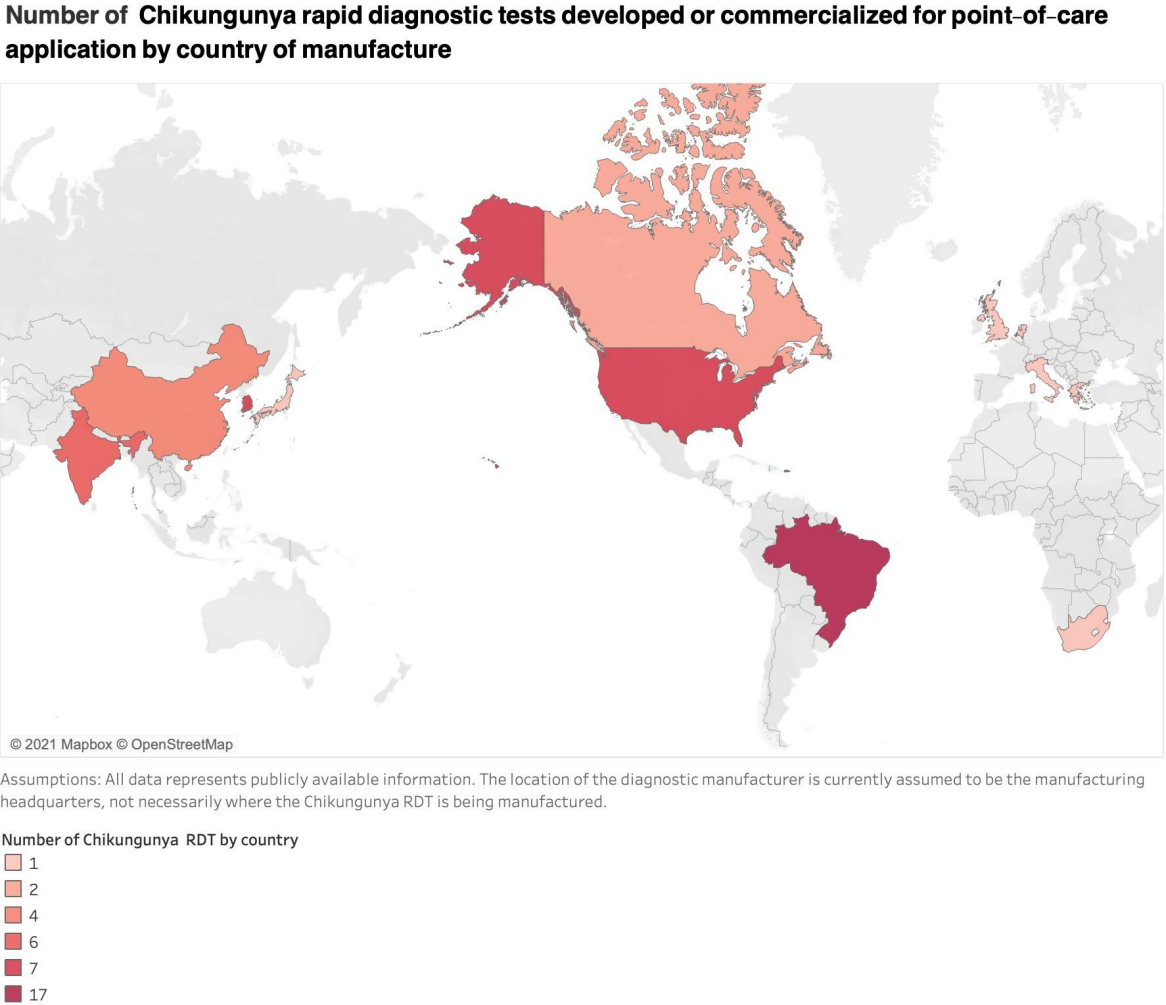
Number of CHIKV RDTs developed or commercialized for POC application by country of manufacture. The world map was created, edited, and colored using Microsoft Excel for Mac, version 16.61.1. Public domain link to map base layer used in creating the figure is available: https://commons.wikimedia.org/wiki/File:BlankMap-World.svg. CHIKV, Chikungunya; POC, point-of-care; RDT, rapid diagnostic test.

Table 2 shows the characteristics of CHIKV RDTs developed or commercialized for POC applications. The countries that manufacturer the most CHIKV RDTs were Brazil (*n* = 17), followed by the United States of America (*n* = 7), South Korea (*n* = 7), and India (*n* = 6) (Fig 1).

**Table 2:** Characteristics of Chikungunya rapid diagnostic tests developed or commercialized for point-of-care application.

Manufacturer	Manufacturer country	Product name	Analytes	Quoted accuracy (Sn/Sp)	Storage temperature (°C)	Sample	Format	Sample volume (uL)	Reading time (min)
**ARKRAY**	Japan	E1-Ag test	E1	NA	NA	S	DS	30	15
**Boditech Med**	South Korea	iChroma	IgG/IgM	NA	NA	S, P, W	IC	30	12
**Meridian Bioscience**	USA	TruQuick CHIKV IgG/IgM 40 T	IgG/IgM	IgG: 94.3/97 IgM: 90.3/99.9	2–30	S, P, W	IC	40	15
**Biotest**	China	MedTest Chikungunya ML-02	IgM/IgG	99.9/99.9	2–30	S, P, W	IC	40	15
**Oscar Medicare Pvt**	India	Oscar Chikungunya test	IgM/IgG	NA	2–30	S, P	IC	NA	NA
**Bio Footprints Healthcare Pvt.**	India	Mytest One Step Chikungunya IgM Test kit	IgM	NA	NA	S, P	IC	NA	NA
**LumiQuick Diagnostics**	USA	Chikungunya test kit QuickProfile	IgG/IgM	NA	4–30	S, P, W	IC	NA	NA
**INTERMEDICAL**	Italy	Chikungunya IgM Rapid Test	IgM	96.9/98.6	2–30	S, P, W	IC	50	15
**Neo Nostics**	China	Chikungunya IgG/IgM Rapid test	IgG/IgM	NA	2–30	S, P, W	IC	NA	NA
**Anand Enterprises**	India	Chikungunya IgM One Step	IgM	NA	NA	S, P	IC	NA	NA
**BIOZEK Medical**	Netherlands	Chikungunya IgG/IgM Rapid Test Cassette	IgG/IgM	NA	NA	S, P, W	IC	NA	NA
**Atlas Link Technology Co.**	China	NOVAtest Chikungunya IgG/IgM Rapid Test Cassette	IgG/IgM	NA	NA	S, P, W	IC	NA	15
**SD BIONSENSOR**	South Korea	STANDARD Q Chikungunya IgM/IgG	IgM/IgG	IgM: 100/97.6 IgG: 100/99.6	2–40	S, P, W	IC	10	15–20
**SD BIONSENSOR**	South Korea	STANDARD F Chikungunya IgM/IgG FIA	IgM/IgG	NA	2–30	S, P, W	FIA		15
**SD BIONSENSOR**	South Korea	STANDARD Q Arbo Panel I (Z/D/C/Y)	IgM (ZIKV, DENV, CHIKV, YFV), DENV NS1	NA	2–40	S, P, W	IC	10–100	15–20
**Tulip Diagnostics**	India	INSIGHT Chikv	IgM		4–30	S, W		5–10	15
**Biopanda Reagents**	UK	Chikungunya IgG/IgM Rapid Test	IgG/IgM	IgG: 94.3/97 IgM: 90.3/99.9	2–30	S, P, W	IC		15
**GenBody**	South Korea	Chikungunya IgM/IgG	IgM/IgG	IgM: 97.1/98.5 IgG: 98/98	2–30	S, P, W	IC	30–60	15–20
**BHAT Bio-Scan**	India	Chikungunya IgM Spot Test	IgM	NA	2–8	S, P	IC	NA	15
**Acro Biotech**	USA	Immunoassay Ivd Chikungunya Rapid Diagnostic Test kit	IgG, IgM	NA	NA	S, P, W	IC	NA	NA
**J. Mitra & Co. Pvt.**	India	Advantage Chikungunya IgM Card	IgM	97.5/99.1	2–30	S, P, W	IC	70	15
**JP BioGen Diagnostics**	Greece	Chikungunya IgM TES	IgM	97.1/91.1	NA	S, P, W	IC	50–100	10
**ICT Diagnostics**	South Africa	Chikungunya IgG/IgM Rapid Test Cassette	IgG/IgM	IgG: 94.3/97 IgM: 90.3/99.9	2–40	S, P, W	IC	40	15
**Diagnostic Automation/Cortez Diagnostics**	USA	OneStep Chikungunya IgG/IgM Combo RapiCard InstaTest	IgG, IgM	NA	4–30	S, P, W	IC	5	15
**HWTAi BioTec**	China	Rapid chikungunya test	IgM	NA	NA	S, P, W	IC	NA	NA
**Teco Diagnostics**	USA	Chikungunya IgM	IgM	NA	NA	S, P, W	IC	NA	NA
**Biocan Diagnostics**	Canada	Chikungunya IgG/IgM Ab Rapid Test	IgG/IgM	NA	NA	S, P, W	IC	NA	NA
**Biocan Diagnostics**	Canada	Zika IgG/IgM Ab, Dengue IgG/IgM & NS1 Ag & Chikungunya IgG/IgM Ab Combo Test	IgM, IgG, NS1	NA	NA	S, P, W	IC	NA	NA
**Bioditech Med**	South Korea	ichroma CHIKV IgG/IgM	IgG, IgM	NA	NA	S, P, W	IC	30	12
**Standard Diagnostics**	South Korea	SD Bioline Chikungunya IgM	IgM	97.1/98.9	1–30	S, P, W	IC	50–100	10
**CTK Biotech**	USA	OnSite Chikungunya IgM Combo Rapid Test	IgM	90.4/98	2–30	S, P, W	IC	5	15
**Chembio Diagnostics**	USA	DPP Chikungunya IgM/IgG assay	IgM/IgG	NA	2–30	S, P, W	IC	10	15
**Bio-Manguinhos**	Brazil	DPP ZCD IgM/IgG	IgM,IgG	IgM: 100/99.4 IgG: 100/100	2–30	S, P, W	IC	10	15
**Orange Life**	Brazil	OL Combo Chikungunya /NS1	DENV NS1/CHIKV IgM	NS1:92.8/98.4 IgM: 98.5/99.5	NA	S, P, W	IC	NA	15–20
**Orange Life**	Brazil	OL Combo Chikungunya Dengue -IgG/IgG	DENV IgM/IgG CHIKV IgM	DENV IgM/IgG: 99.5/98.5 CHIKV IgM: 98.5/99.5	NA	S, P, W	IC	NA	15–20
**Orange Life**	Brazil	OL Chikungunya IgM	IgM	98.5/99.5	NA	S, P, W	IC	NA	15–20
**Orange Life**	Brazil	OL Chikungunya IgG/IgM	IgM/IgG	IgM/IgG: 98.5/98.5	NA	S, P, W	IC	NA	15–20
**Eco Diagnostica**	Brazil	Chikungunya IgG/IgM ECO Test	IgG/IgM	IgG: 100/99.6 IgM: 100/97.6	2–30	S, P, W	IC	10	15
**Eco Diagnostica**	Brazil	Chikungunya IgM ECO Test	IgM	90.3/100	2–30	S, P, W	IC	30–45	15
**Bahiafarma**	Brazil	Chikungunya IgM RDT	IgM	94/95	2–30	S, P, W	IC	30–60	10
**Ebram Ltda.**	Brazil	Chikungunya IgG/IgM	IgG/IgM	IgG: 94.3/97 IgM: 90/99.9	2–30	S, P, W	IC	40	15–20
**WAMA Diagnostica**	Brazil	Immuno-Rapido Chikungunya IgG/IgM	IgG/IgM	IgG: 100/99.3 IgM:100/97.9	2–30	S, P, W	IC	10	15–20
**Biocon diagnosticos**	Brazil	Chikungunya Test (IgG/IgM)	IgG/IgM	IgG: 94.3/97 IgM: 90.3/99	NA	S, P	IC	NA	15
**Biocon diagnosticos**	Brazil	Chikungunya IgM Test	IgM	96.6/98	NA	W	IC	NA	15

DS, dipstick; FIA, fluorescent immunoassay; IC, immunochromatographic assay; NA, not available; P, plasma; S, serum; Sn/sp, sensitivity/specificity; W, whole blood.

Overall, the CHIKV RDT market is fragmented, but the manufacturer with the most products in the market is Chembio Diagnostics Brazil (*n* = 5 products) and SD BIOSENSOR (*n* = 3 products) (S2 Fig). Almost all assays are antibody-based RDTs (*n* = 43) designed in an immunochromatographic format. There were neither antigen-based RDTs nor a combination of antibody and antigen-based RDTs commercially available. Our searches for approved assays in national regulatory authorities did not find any assay registered by the European Medicines Agency or the US Food and Drug Administration. Conversely, the Brazilian National Health Surveillance Agency (ANVISA) has approved 23 CHIKV RDTs for clinical use. Of these, 5/23 (21.7%) were multiplex assays with targets concomitant for Dengue and Zika analytes. S1 Table shows the characteristics of CHIKV RDTs approved by the ANVISA.

### Diagnostic accuracy results

Table 3 shows a summary of the diagnostic assessments included conducted between 2005 and 2018. In total, 3,222 samples were tested with RDTs across all the studies (S3 Fig). Sample types included whole blood, plasma, and serum. Eleven studies examined the performance of antibody-based RDTs [9,11–19], while 5 the antigen-based RDTs [5,6,20–22].

**Table 3:** Summary of diagnostic assessments of Chikungunya antibody or antigen-based rapid diagnostic tests, 2005–2018.

Assay	Study [reference]	Year	Sample size	Time from symptom onset to testing (days)	Reference comparator	Analyte target	Sensitivity (95% CI)	Specificity (95% CI)
**Antibody-based RDT**								
**ichroma Chikungunya virus (IgG/IgM)**								
	Lee H and colleagues 2020 [11]	ND	256	ND	Inbios IgM/IgG ELISA Euroimmun IgM/IgG ELISA	IgM IgG	100 (94.7–100) 100 (92.4–100)	99.4 (97.5–99.4) 100 (98.3–100)
**Chikungunya IgM/IgG (GenBody)**								
	Kim WS and colleagues 2019 [12]	2014	770	ND	ELISA RT-PCR	IgM IgG	83 100	97 100
**Multiplex RDT (under development)**								
	Wang R and colleagues 2019 [19]	ND	50	ND	Euroimmun ELISA	IgM IgG	83 100	97 100
**OnSite Chikungunya IgM Rapid Test**								
	Burdino E and colleagues 2016 [13]	2014–2015	8	7–30	Euroimmun IgM/IgG IFA RT-PCR	IgM	37.5	100
	Prat CM and colleagues 2014 [14]	2005–2014	23	ND	In-house IgM/IgG ELISA In-house neutralization test	IgM	20	93
	Kosasih H and colleagues 2012 [15]	ND	132	1 to ≥21	In-house IgM ELISA RT-PCR	IgM	20.5	100
	Arya SC and colleagues 2011 [16]	2010	100		IgM ELISA	IgM	35.7	NA
	Yap G and colleagues 2010 [17]	2008	225	3.75 to >7	IgM IFA In-house IgM ELISA RT-PCR	IgM	12.1	100
	Mistretta M and colleagues 2009 [9]	2006–2008	116	ND	Euroimmun IFA	IgM	85	95
	Johnson BW and colleagues 2016 [18]	ND	27	2–33	CDC in-house MAC-ELISA	IgM	13.04 (2.78–33.59)	100 (39.76–100)
**SD Bioline Chikungunya IgM test**								
	Prat CM and colleagues 2014 [14]	2005–2014	23	ND	In-house IgM/IgG ELISA In-house neutralization test	IgM	30	73
	Kosasih H and colleagues 2012 [15]	ND	132	1 to ≥21	In-house IgM ELISA RT-PCR	IgM	50.8	89.2
	Rianthavorn P and colleagues 2010 [4]	2008	527	1 to ≥14	SD Bioline IgM ELISA RT-PCR	IgM	37	85
	Johnson BW and colleagues 2016 [18]	ND	31	2–33	CDC in-house MAC ELISA	IgM	0	100 (59–100)
**Antigen-based RDT**								
**E1-Antigen test**								
	Huits R and colleagues 2018 [6]	2006–2014 2014–2015	98	≤10	ECSA and Asian genotype CHIKV-specific RT-PCR Euroimmun IgM/IgG IFA	E1-antigen	88.9 (56.5–98) for the ECSA genotype 33.3 (19.2–51.2) for the Asian genotype	83.1 (71.5–90.5)
	Okabayashi T and colleagues 2015 [20]	2008–2013	112	1–14	ECSA, Asian, and West African genotype CHIKV-specific RT-PCR Nova Tec IgM ELISA	E1-antigen	91.2 for the ECSA genotype 89.4 for the overall genotypes	93.8 for the ECSA genotype 94.4 for the overall genotypes
	Jain J and colleagues 2018 [21]	2016	123	1–15	IgM ELISA RT-PCR	E1-antigen	93.7	95.5
	Suzuki K and colleagues 2020 [5]	2014–2015 2017–2018	280	≤7	IgM ELISA RT-PCR	E1-antigen	92	100
**E1/E2-lateral flow antigen test**								
	Reddy A and colleagues 2020 [22]	ND	189	1–5	RT-PCR	E1/E2-antigen	62.5–100 for Honduras’ AB combination A 62.5–100 for Honduras’ AB combination B 77.7–100 for Colombia’ AB combination B	92.3–100 for Honduras’ AB combination A 75–100 for Honduras’ AB combination B 85.7–100 for Colombia’s AB combination B

CI, confidence intervals; ECSA, East/Central/South/Africa chikungunya lineage/genotype; ELISA, enzyme-linked immunosorbent assay; IFA, immunofluorescence assay; IgM, immunoglobulin M; IgG, immunoglobulin G; IQR, interquartile range; ND, not defined; RT-PCR, reverse transcription polymerase chain reaction.

**Fig 2:**
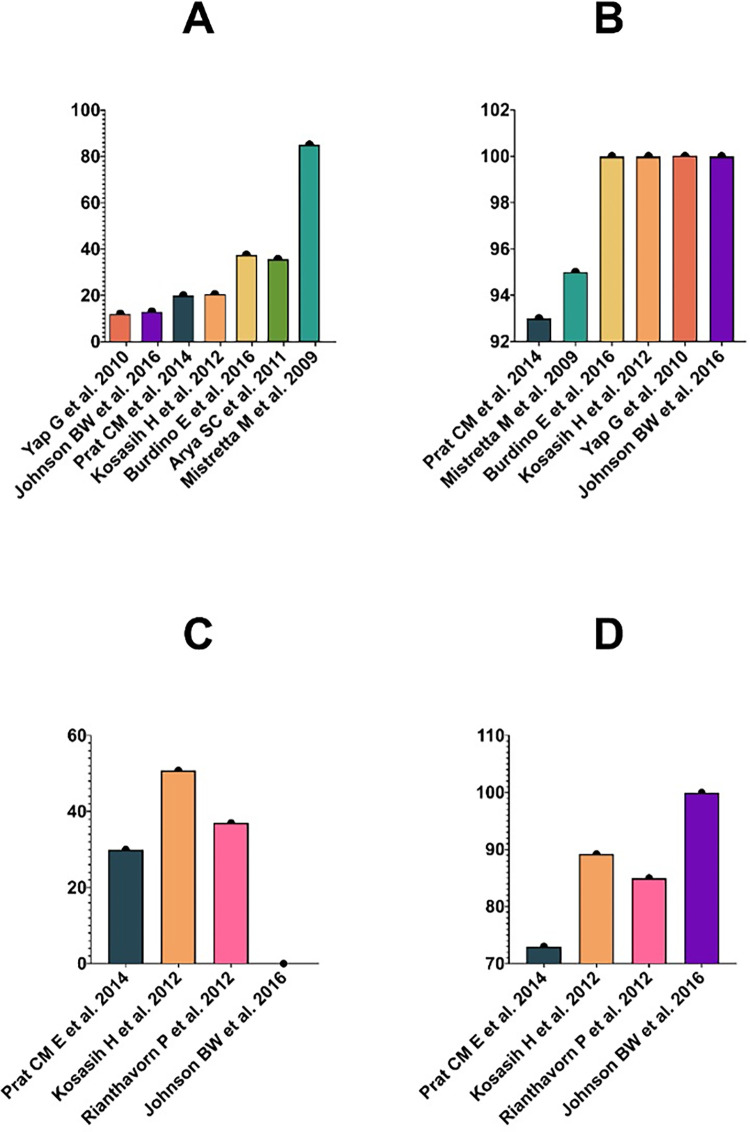
Summary of diagnostic accuracy studies evaluating the OnSite Chikungunya IgM Combo Rapid test (CTK Biotech, Poway, CA, USA) and the SD BIOLINE Chikungunya IgM test (Standard Diagnostics, Yongin-si, South Korea).

The predominant CHIKV RDT assay evaluated in the studies was the OnSite Chikungunya IgM Combo Rapid test (CTK Biotech, Poway, CA, USA) in 8/16 (50%) studies, followed by the SD BIOLINE Chikungunya IgM test (Standard Diagnostics, Yongin-si, South Korea) in 3/16 (18.7%) studies. The most of antibody RDTs studies target IgM, while 3 studies target both IgM and IgG immunoglobulin components. Fig 2 shows the diagnostic accuracy for the OnSite Chikungunya IgM Combo Rapid test and SD BIOLINE Chikungunya IgM test.

Overall, the sensitivity of the RDT IgM component typically ranged between 20% and 100%. The sensitivity of the RDT IgG component was 100%. The RDT IgM specificity ranged from 73% to 100%, and the IgG specificity was 100%. Interestingly, some studies reported an increase in the overall sensitivity of antibody-based RDT over time [4,15].

There are 2 types of antigen-based RDTs evaluated—E1 and E1/E2-antigens tests. The sensitivity of the E1-antigen tests ranged from 33.3% to 100%. Conversely, the specificity varied between 83.1% and 100%.

### Risk of bias assessment

**Fig 3:**
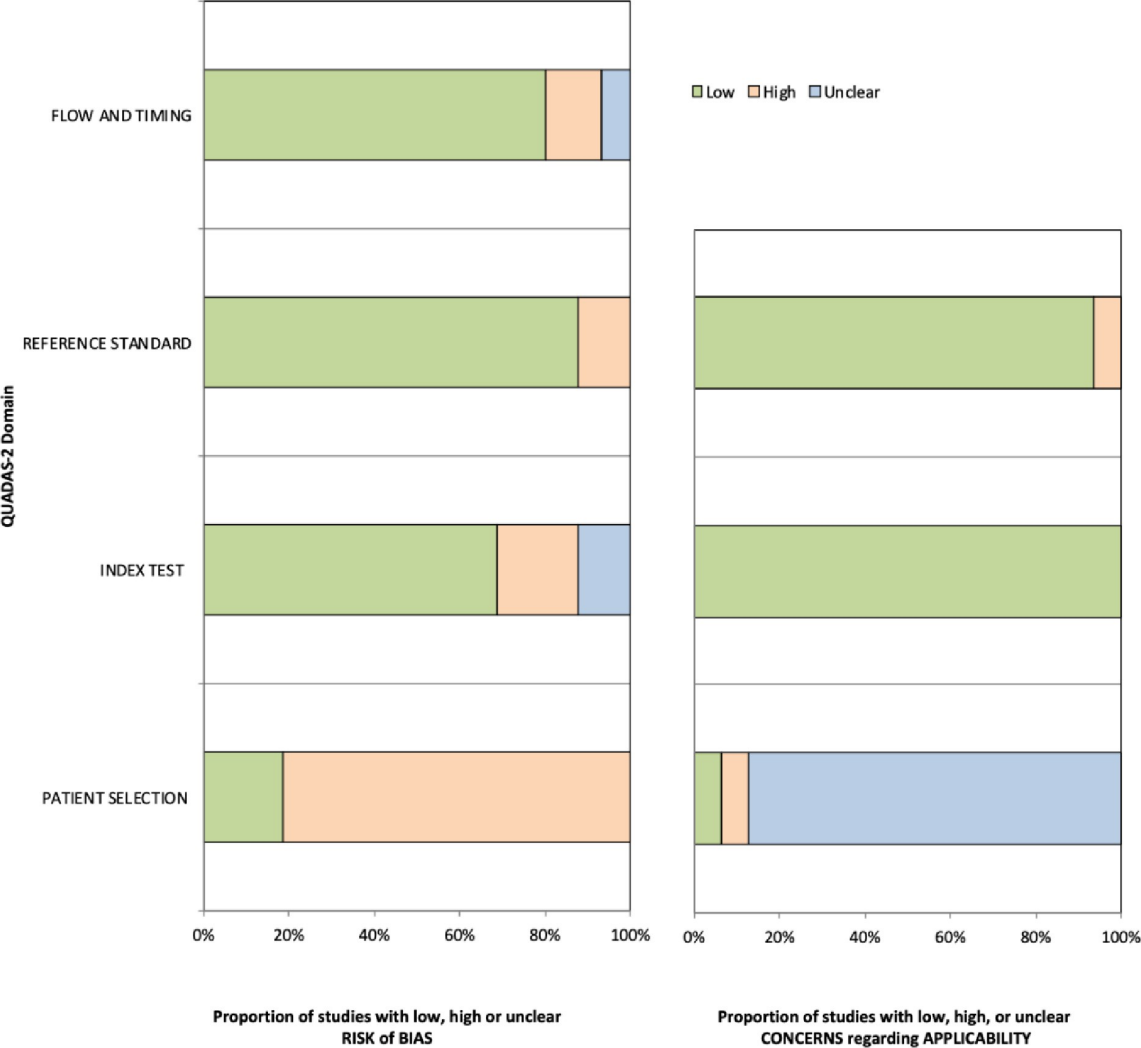
QUADAS-2 assessment of studies.

Fig 3 summarizes the QUADAS-2 assessment by study. There were patient selection applicability concerns for most of the study (*n* = 14) because there was a lack of sufficient information reported in the studies regarding the patient population, demographic features, setting of the study, or presence of comorbidities. Similarly, there was a high risk of bias in the patient selection domain because only 2 studies enrolled a consecutive or random sample of eligible patients with suspicion of CHIKV infection to reduce the bias in the diagnostic accuracy of the index test.

## Discussion

### Summary of evidence

This scoping review identified 17 studies conducted between 2005 and 2018, addressing the research stage on CHIKV RDTs across various settings. Our findings indicate a paucity of research focusing on field trials and implementation studies related to CHIKV RDTs. Our work provides a global view of publicly available data on CHIKV RDTs currently under development or commercially available. We also found that the in vitro diagnostic medical device manufacturers are primarily concentrated on CHIKV antibody RDTs, and their accuracy overall performs poorly and should not be used in clinical settings as long as they suffer significant improvements [4,15]. Conversely, antigen RDTs, although still in a development phase, promise to have a high level of sensitivity and specificity across the distinct CHIKV genotypes [5,21].

Given the problems associated with the existing diagnostic strategies for CHIKV, there is a clear and urgent need for new, appropriate diagnostic tools for CHIKV that meet the ideal product profile of “REASSURED” diagnostics [23]. The characteristics of the diagnostics products mentioned above are defined by a set of criteria that includes: (i) real-time connectivity; (ii) ease of specimen collection; (iii) environmental friendliness; (iv) affordable by those at risk of infection; (v) sensitive (few false-negatives); (vi) specific (few false-positives); (vii) user-friendly (simple to perform and requiring minimal training); (viii) rapid (to enable treatment at first visit) and robust (does not require refrigerated storage); (xi) equipment-free; and (x) delivered to those who need it. Few products right now meet the ideal “REASSURED” profile, and new research and investments are required to develop those that match the profile needed. Pertinent questions about feasibility, acceptability, cost-effectiveness, sustainability, and policy implications must be addressed before the widespread use of CHIKV RDTs in endemic countries. More importantly, we also need to address the impact of CHIKV RDTs into integrated fever case management and how its implementation translates into a better prescription practice for acute febrile patients (i.e., reducing unnecessary antibiotic prescription).

**Fig 4:**
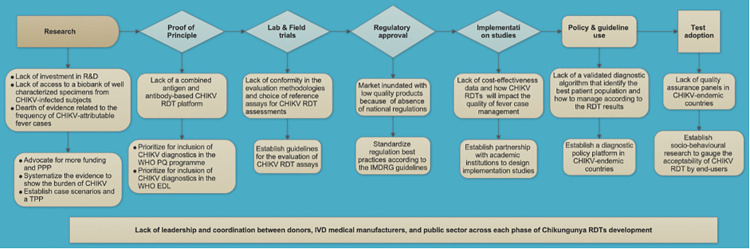
CHIKV RDTs: Fragmented landscape presents market challenges and opportunities for interventions. CHIKV, Chikungunya; RDT, rapid diagnostic test; WHO, World Health Organization.

The CHIKV RDTs diagnostic landscape is fragmented, with many gaps along the development pathway. Fig 4 shows our proposed conceptual framework that delineates the challenges and opportunities across each stage of CHIKV RDT development. Concerted efforts leading by different stakeholders (i.e., international donors, industry, public sector, and end-users) should be put together to bring more equity to the availability of appropriate CHIKV RDTs to those needed most.

### Limitations

Our work has limitations. Although we made a herculean effort to identify the highest numbers of CHIKV RDTs manufactured or commercially available in the market, we understand that some could not be identified and were not publicly available. However, we addressed this bias by looking into CHIKV RDTs that national/regional regulatory agencies have approved or those that provided data from unpublished sources (i.e., conference abstracts, manufacturers’ reports). Next, we did not provide an effect estimate for the results of diagnostic accuracy studies, because as shown in our risk of bias assessment, the studies included were very heterogeneous, and a meta-analytic approach would be useless.

## Conclusions

**Fig 5:**
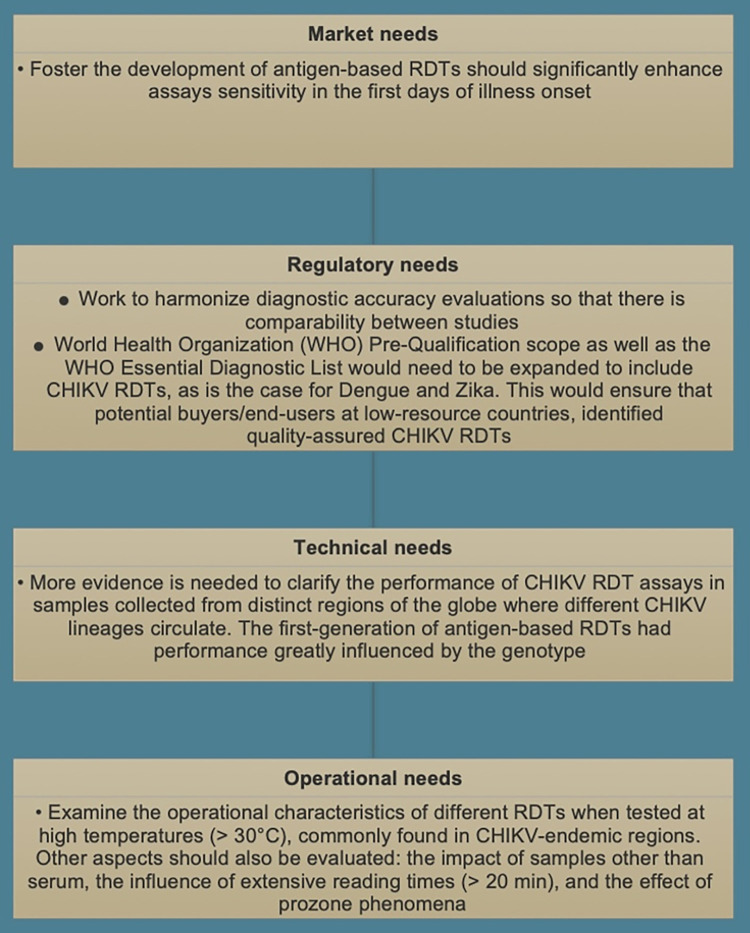
Future needs of immunoassay-based rapid diagnostic tests for CHIKV infection. CHIKV, Chikungunya; RDT, rapid diagnostic test; WHO, World Health Organization.

Our scoping review demonstrated substantial gaps in the current diagnostic landscape of CHIKV RDTs. The future needs of immunoassay-based RDTs for CHIKV are summarized in Fig 5.

The time is suitable for a collaborative, focused initiative between policy-makers and other relevant stakeholders to address the urgent need for new, appropriate CHIKV RDTs. Unprecedented opportunities for market interventions exist and utilize new technologies to make a significant, measurable impact. Further research is desperately needed to facilitate the incorporation of CHIKV RDTs into integrated fever algorithms, and socio-behavioral research should be done to evaluate end-user acceptability.

**Key learning points**

- Chikungunya is an emerging viral disease with outbreak potential.
- Access to timely, accurate diagnostics is fundamental to equitable and effective healthcare provision.
- The global landscape of chikungunya rapid diagnostic tests is fragmented and heavily depended on antibody rapid tests, which had a poor diagnostic performance.
- Addressing shortfalls in chikungunya rapid diagnostic testing must be an urgent priority and antigen rapid tests promise to reduce diagnostic gaps and improve access.
- Strong country leadership is needed to accelerate investment in research and product development and expand manufacturing capacity for diagnostics and surveillance.

**Top five papers**

1. Fleming K, Horton S, Wilson M, Atun R, DeStigter K, Flanigan J, et al. The Lancet Commission on diagnostics: transforming access to diagnostics. Lancet. 2021;398(10315):1997–2050.
2. Suzuki K, Huits R, Phadungsombat J, Tuekprakhon A, Nakayama EEEE, Van Den Berg R, et al. Promising application of monoclonal antibody against chikungunya virus E1-antigen across genotypes in immunochromatographic rapid diagnostic tests. Virol J. 2020;17:90.
3. Reddy A, Bosch I, Salcedo N, Herrera BB, de Puig H, Narváez CF, et al. Development and Validation of a Rapid Lateral Flow E1/E2-Antigen Test and ELISA in Patients Infected with Emerging Asian Strain of Chikungunya Virus in the Americas. Viruses. 2020;12.
4. Land KJ, Boeras DI, Chen X-S, Ramsay AR, Peeling RW. REASSURED diagnostics to inform disease control strategies, strengthen health systems and improve patient outcomes. Nat Microbiol. 2019;4:46–54.
5. Johnson BW, Goodman CH, Holloway K, De Salazar PM, Valadere AM, Drebot MA. Evaluation of commercially available Chikungunya Virus Immunoglobulin M detection assays. Am J Trop Med Hyg. 2016;95:182–92.

## Supporting information

S1 PRISMA checklist Preferred Reporting Items for Systematic reviews and Meta-Analyses extension for Scoping Reviews (PRISMA-ScR) Checklist. (DOCX)

S1 PRISMA Flowchart PRISMA flowchart diagram. (TIFF)

S1 Fig Sources of Chikungunya samples evaluated for rapid diagnostic test, 2005–2018. The world map was created, edited, and colored using Microsoft Excel for Mac, version 16.61.1. Public domain link to map base layer used in creating the figure is available: https://commons.wikimedia.org/wiki/File:BlankMap-World.svg.
(TIFF)

S2 Fig Global Chikungunya rapid diagnostic tests landscape—key players on industry, 2005–2018. (TIF)

S3 Fig Number of samples tested according to Chikungunya rapid diagnostic test, 2005–2018. (TIFF)

S1 Table Characteristics of commercial Chikungunya rapid diagnostic tests for point-of-care application registered by the Brazilian National Health Surveillance Agency. (DOCX)
