# Supplementary figures and images for: Mapping the global landscape of chikungunya rapid diagnostic tests: A scoping review
Source: PLoS Negl Trop Dis. 2022 Jul 25;16(7):e0010067. doi: 10.1371/journal.pntd.0010067 (PMC9352193; doi:10.1371/journal.pntd.0010067)

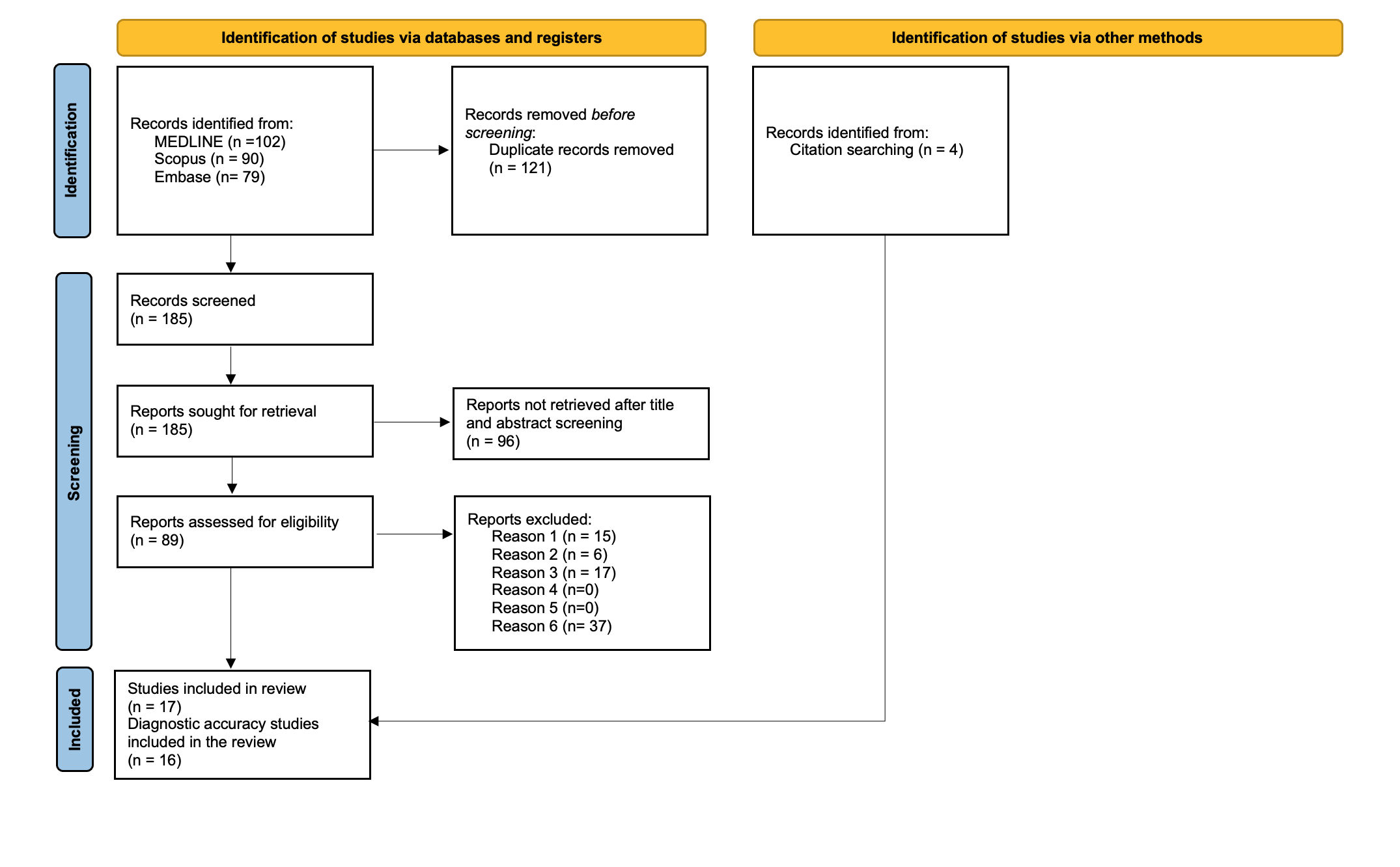

Supplement: S1 PRISMA Flowchart — (TIFF) [file pntd.0010067.s002.tiff]

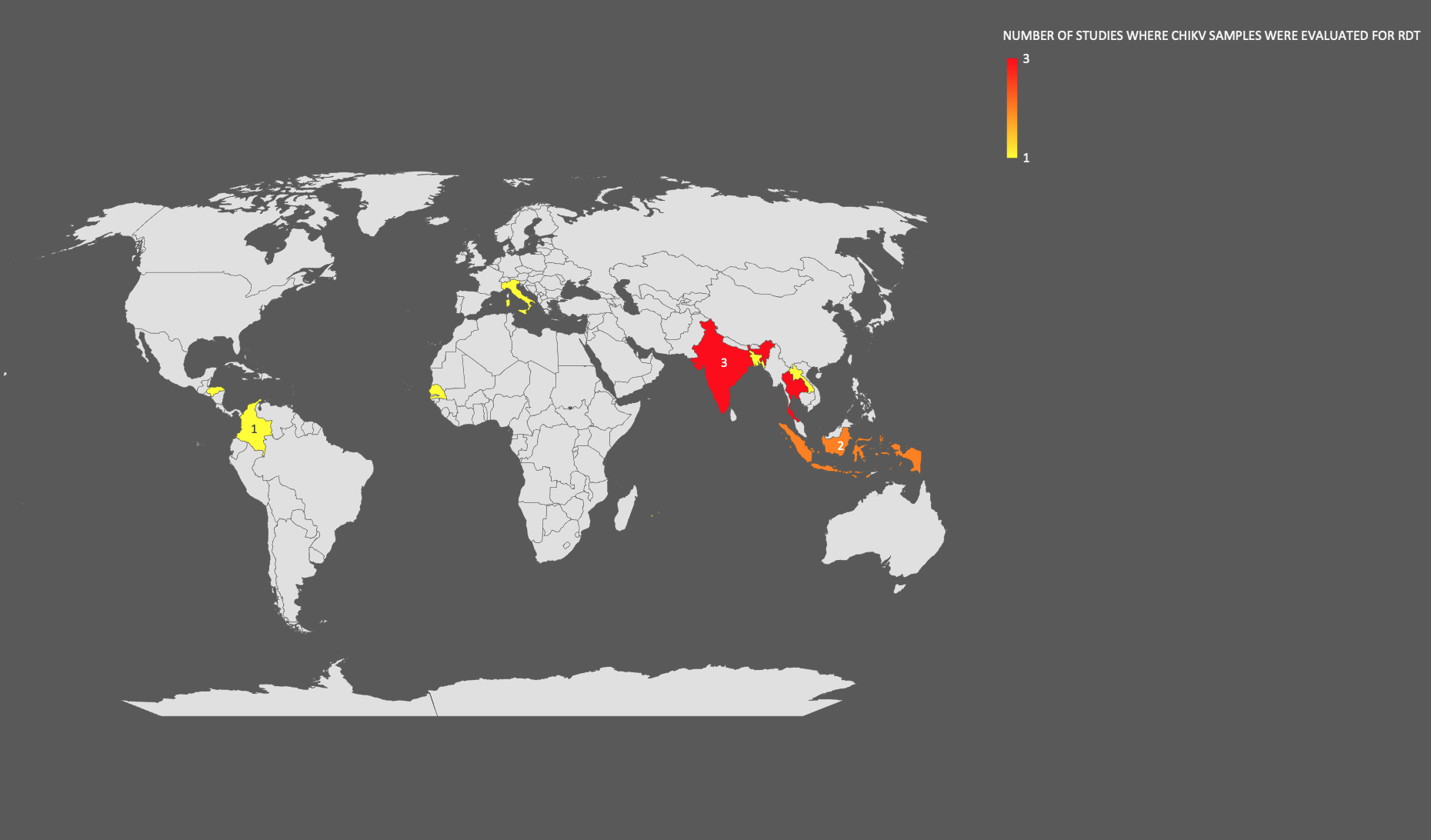

Supplement: S1 Fig — The world map was created, edited, and colored using Microsoft Excel for Mac, version 16.61.1. Public domain link to map base layer used in creating the figure is available: https://commons.wikimedia.org/wiki/File:BlankMap-World.svg. (TIFF) [file pntd.0010067.s003.tiff]

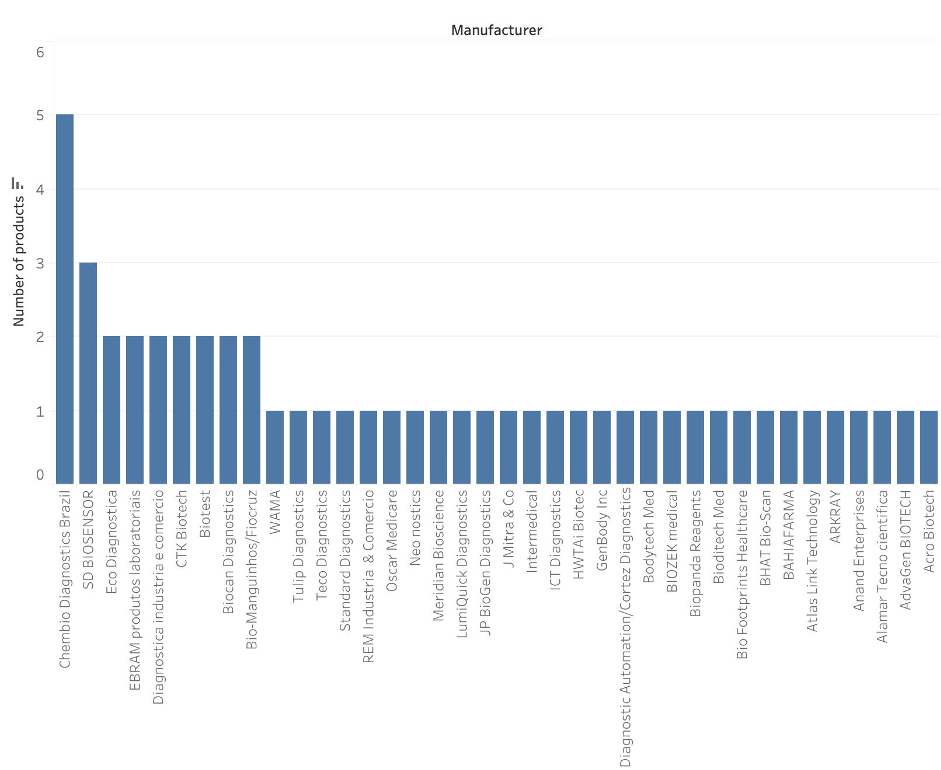

Supplement: S2 Fig — (TIF) [file pntd.0010067.s004.tif]

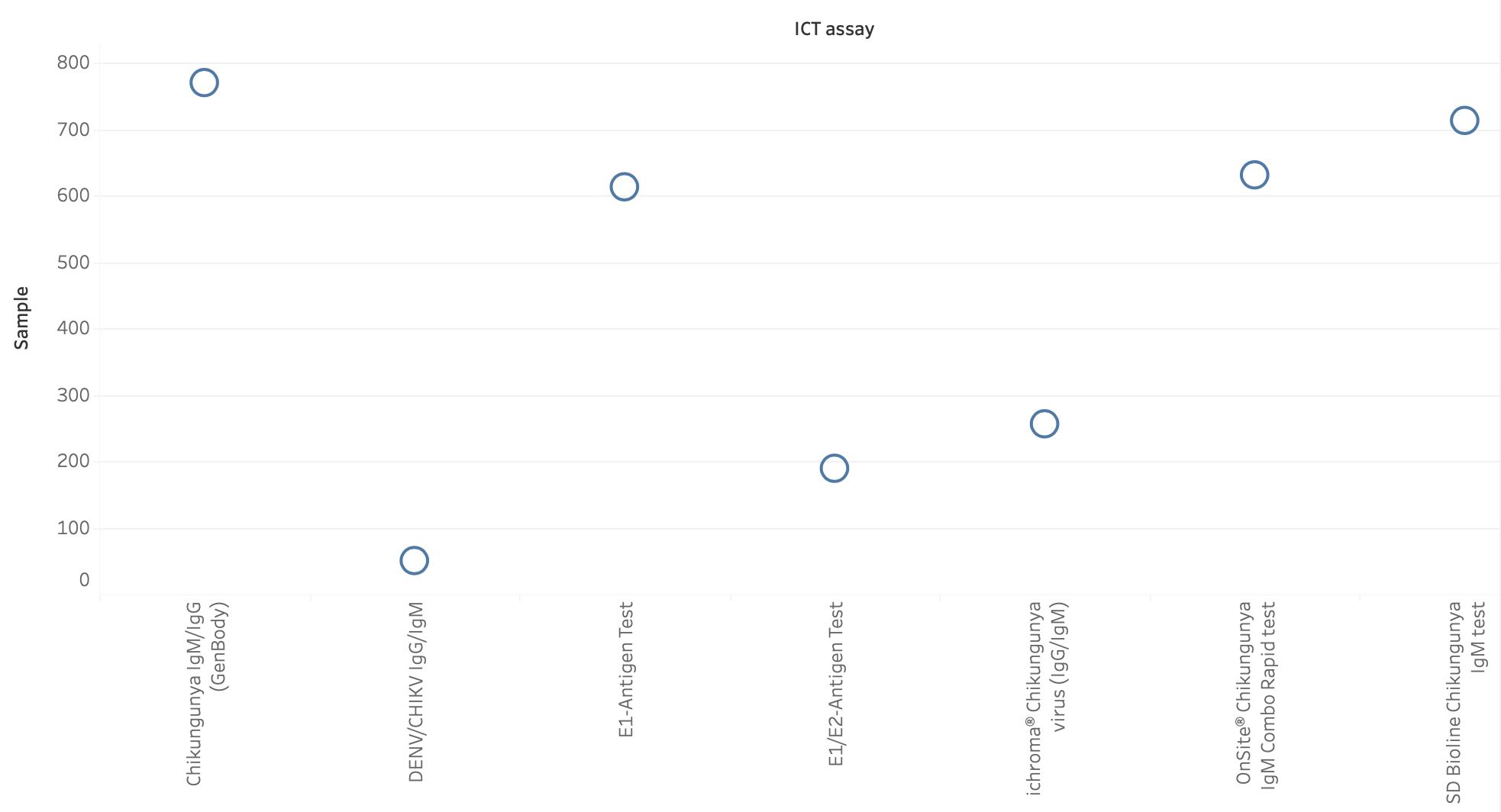

Supplement: S3 Fig — (TIFF) [file pntd.0010067.s005.tiff]
